# Implant survival and risk factors for failure after proximal femoral megaprosthetic reconstruction

**DOI:** 10.1051/sicotj/2025031

**Published:** 2025-08-26

**Authors:** Anastasios G. Roustemis, Panayiotis Gavriil, Stavros Goumenos, Ioannis Trikoupis, Vasileios Karampikas, Panagiotis Koulouvaris, Vasileios Kontogeorgakos, Olga Savvidou, Andreas F. Mavrogenis, Panayiotis J. Papagelopoulos

**Affiliations:** 1 First Department of Orthopaedics, National and Kapodistrian University of Athens, School of Medicine Rimini Street Chaidari 124 62 Athens Greece; 2 Department of Orthopaedics, Charité – Universitätsmedizin Berlin 20 Schumannstraße 10117 Berlin Germany; 3 The Royal Orthopaedic Hospital NHS Foundation Trust, Royal Orthopaedic Hospital Bristol Road South Northfield Birmingham B31 2AP United Kingdom

**Keywords:** Risk factors, Limb salvage, Survival, Proximal femoral megaprosthetic reconstruction, Complications

## Abstract

*Background*: Proximal femoral megaprosthetic reconstruction is a well-established solution for extensive bone loss in the hip region. Despite its utility in limb salvage, it carries notable complication rates, reported between 30% and 40%, along with increased morbidity and mortality. This study evaluated implant and patient survival, failure modes, and associated risk factors. *Methods*: We retrospectively reviewed 165 patients who underwent proximal femoral megaprosthetic reconstruction between 2003 and 2023. Indications included primary bone tumors (*n* = 67), metastatic bone disease (*n* = 60), and non-oncologic conditions (*n* = 38). A total of 57 METS (Stanmore) and 108 MUTARS (Implantcast) implants were used. Median follow-up was 5 years (range: 0.25–17 years). *Results*: Mean implant survival was 5.13 years (range: 0.2–17 years), with an overall complication rate of 30.9%. The most common failure modes were type 1 (11.5%) and type 4 (13.3%) per Henderson classification. Five-year implant survival ranged from 60% to 70% across indications. Independent risk factors for type 4 failure included prolonged hospitalization (OR = 1.07, *p* = 0.020) and longer operative time (OR = 1.01, *p* = 0.023). Silver-coated implants showed a trend toward reduced infection (OR = 0.18, *p* = 0.29), though not statistically significant. METS implants were associated with lower type 1 failure risk (OR = 0.09, *p* = 0.020), with a soft-tissue failure rate of 3.5% versus 15.7% for MUTARS. *Conclusion*: Proximal femoral megaprostheses remain effective for limb salvage but are linked to a substantial complication burden. Recognition of modifiable and patient-specific risk factors may improve surgical outcomes and reduce failure rates.

## Introduction

Proximal femoral megaprosthetic reconstruction is a limb-salvage technique for managing extensive bone loss due to malignancy, complex trauma, or failed arthroplasty [1–3]. Since 1943, the technique has evolved into a standard of care for primary bone sarcomas and metastatic lesions, supported by advances in imaging, chemotherapy, and prosthetic design [4, 5].

Modern megaprostheses provide immediate stability and early mobilization [1], but their use remains technically demanding and associated with high complication rates [2, 6, 7]. Despite widespread use in both oncologic and non-oncologic settings, prior studies are often limited by narrow sample populations, heterogeneous methodologies, or incomplete reporting of implant-specific risk factors, failure mechanisms, and long-term outcomes [8, 9].

This study evaluates implant and patient survival, failure mechanisms, and risk factors following proximal femoral megaprosthetic reconstruction over 20 years at a high-volume orthopedic center, to better inform clinical decisions in both oncologic and non-oncologic settings.

## Materials and methods

We retrospectively reviewed the medical records of 165 patients who underwent proximal femoral megaprosthetic reconstruction at the authors’ institution between 2003 and 2023. There were 91 men (55.2%) and 74 women (44.8%), with a mean age of 61.8 years (range: 24–89 years). Surgical indications included 67 patients (40.6%) with primary bone tumors, 60 (36.4%) with metastatic disease, and 38 (23.0%) with non-oncologic conditions (designated as Indications 1, 2, and 0, respectively) (Tables 1 and 2). Patients lost to follow-up within 2 years (*n* = 11), those with incomplete clinical or imaging records (*n* = 6), or those without documented informed consent (*n* = 3) were excluded. All patients or their legal representatives provided written informed consent. The study was approved by the Institutional Review Board of the authors’ institution.

**Table 1:** Summary of patient and implant characteristics.

Category	Number of patients
Demographics	
Total patients	165
Male	91
Female	74
Indications	
Primary bone tumors	67
Metastatic disease	60
Non-oncologic indications	38
Implant type	
MUTARS^®^ implants	108
Stanmore prostheses	57
Fixation type	
Cemented fixation	103
Uncemented fixation	62
Silver coating	
Silver-coated implants	63
Non-coated implants	102
Hip reconstruction	
Bipolar femoral head	125
Total hip arthroplasty	40

**Table 2:** Diagnosis and indications of patients undergoing proximal femoral replacement.

Diagnosis	Number of patients
Primary bone tumors (Indication 1)	
Chondrosarcoma	23
Osteosarcoma	11
Ewing sarcoma	6
Leiomyosarcoma	4
Liposarcoma	3
Chondroblastic osteosarcoma	1
Dedifferentiated chondrosarcoma	1
Pleiomorphic sarcoma	1
Total (primary bone tumors)	67
Secondary bone tumors (Indication 2)	
Carcinoma of Breast	10
Carcinoma of kidney	9
Carcinoma of prostate	5
Carcinoma of lung	3
Other metastasis	33
Total (secondary bone tumors)	60
Non-oncologic patients (Indication 0)	
Revision arthroplasty	20
Infection	12
Complex trauma	6
Total (non-oncologic)	38

All patients underwent preoperative biopsy and staging, followed by either wide or palliative resection depending on tumor histology. Chemotherapy and/or radiotherapy were administered when indicated.

Collected variables included demographics, ASA classification, comorbidities, diagnosis, resection specimen size, perioperative details (e.g., transfusion volume, operative time), implant characteristics, and rehabilitation protocols. Implants used included MUTARS^®^ (Implantcast GmbH, Germany) in 65.5% and METS (Stanmore Implants Worldwide) in 34.5% of cases. Implant selection was primarily based on surgeon preference and institutional availability, though certain cases required individualized choice based on anatomical or oncologic considerations. Cemented fixation was employed in 62.4% of implants. Silver-coated components were used in 38.2% of reconstructions. Bipolar femoral heads were used in 75.8% of patients, while 24.2% underwent total hip arthroplasty. Abductor mechanism reconstruction was performed in all cases. Postoperative rehabilitation consisted of 3–4 weeks of protected weight-bearing.

Follow-up assessments were scheduled monthly for the first 3 months, quarterly for 2 years, semiannually until year 5, and annually thereafter. Outcome measures included implant and patient survival, complication rates, amputation, and functional assessment using the Musculoskeletal Tumor Society (MSTS) scoring system [10]. Complications were classified according to the Henderson classification [11], and periprosthetic infection was diagnosed using clinical, laboratory, and intraoperative criteria.

Statistical analyses included descriptive statistics, Kaplan–Meier survival estimates, log-rank tests, multivariate logistic regression for complication risk factors, and a 1-year landmark analysis for silver-coated implants. No a priori power analysis was performed due to the retrospective nature of the study and the inclusion of a complete consecutive cohort. A *p*-value < 0.05 was considered statistically significant. All analyses were performed using R software version 4.3.1.

## Results

### Implant survival

**Figure 1:**
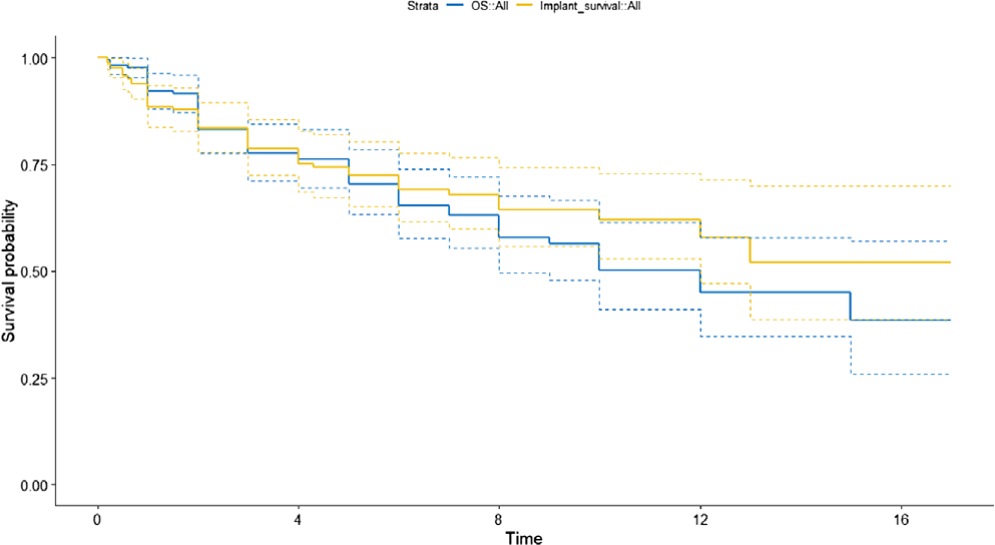
Kaplan–Meier curve comparing implant survival (yellow) and patient survival (blue) across the entire cohort. Five-year implant survival was 69.3%, and five-year patient survival was 70.4%. The parallel trendlines illustrate that most implants remained functional until patient death, particularly in metastatic cases.

Of the 165 implants, the estimated survival was 77.0% at 3 years and 69.3% at 5 years. Stratified by indication: non-oncologic cases (Indication 0) showed 77.0% and 69.3%, primary tumors (Indication 1) 71.3% and 64.2%, and metastatic lesions (Indication 2) 83.7% and 75.3%, respectively (Figure 1). Implant survival closely mirrored patient survival, particularly in cases of metastatic disease, where most implants remained functional until patient death. In contrast, among patients with longer life expectancy, such as those with primary tumors or non-oncologic indications, complications, especially soft-tissue failure and infection, were more likely to lead to early implant failure.

### Patient survival

**Figure 2:**
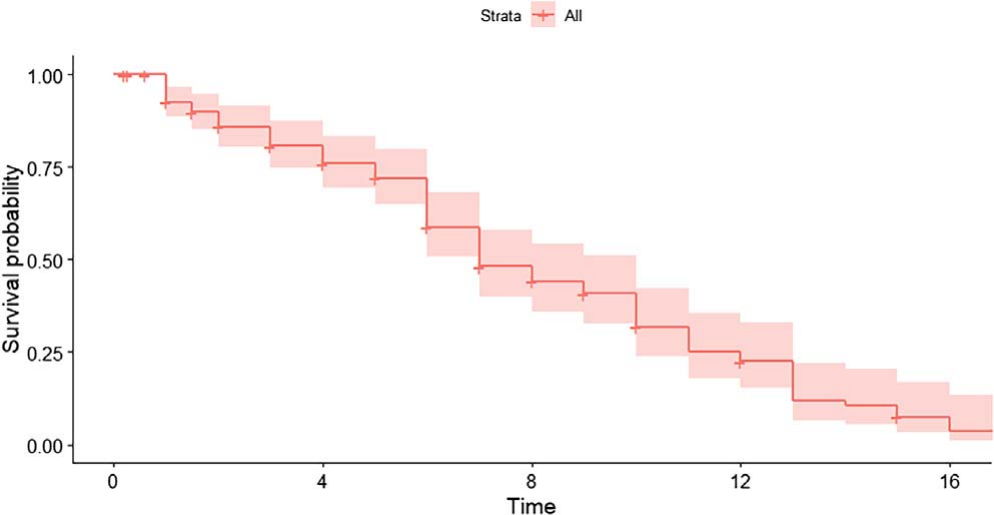
Kaplan–Meier curve for overall patient survival. Survival at 3 and 5 years was 77.6% and 70.4%, respectively (95% CI: 71.2%–84.6% and 63.3%–78.4%). No stratification by surgical indication was applied in this curve.

**Figure 3:**
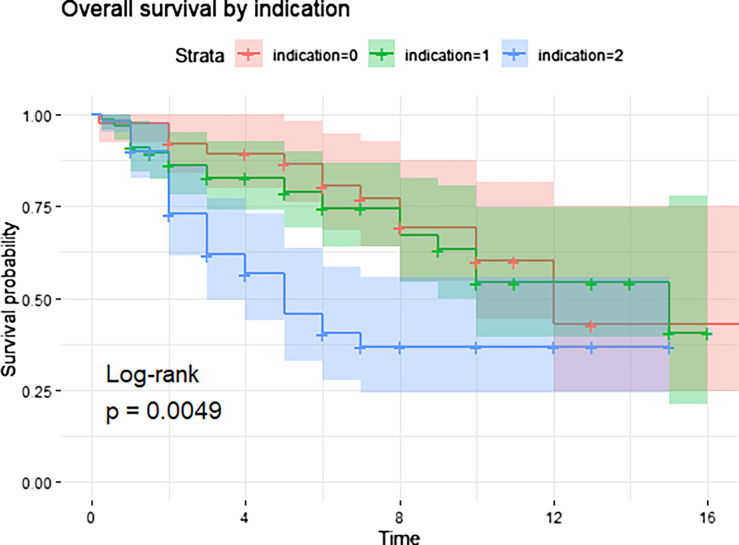
Kaplan–Meier curve showing patient survival stratified by indication: metastatic disease (Indication 2, blue) – 83.7% at 3 years and 75.3% at 5 years; non-oncologic (Indication 0, red) – 77.0% and 69.3%; primary bone tumors (Indication 1, green) – 71.3% and 64.2%. The difference between groups was statistically significant (log-rank *p* = 0.0049).

Overall patient survival was 77.6% at 3 years (95% CI: 71.2–84.6%) and 70.4% at 5 years (95% CI: 63.3–78.4%) (Figure 2). By indication: Indication 0 – 77.0% and 69.3%, Indication 1 – 71.3% and 64.2%, Indication 2 – 83.7% and 75.3% (Figure 3). Relative risks (RR) for complications (vs. Indication 0): 1.08 (95% CI: 0.58–2.00) for Indication 1, and 0.92 (95% CI: 0.48–1.77) for Indication 2.

### Complications and modes of failure

The overall complication rate was 30.9% (51/165). According to the Henderson classification, the most frequent failure modes were: Type 1 (soft tissue) – 11.5%, Type 2 (aseptic loosening) – 1.8%, Type 3 (structural) – 2.4%, Type 4 (infection) – 13.3%, and Type 5 (local recurrence) – 1.8%. Fifteen implants (9.1%) required revision, and nine patients (5.5%) underwent amputation. The median MSTS score was 22/30. Subgroup analysis of MSTS scores by complication type or implant group was not feasible due to incomplete or heterogeneously reported functional data across the cohort.

### Survival and complications

**Figure 4:**
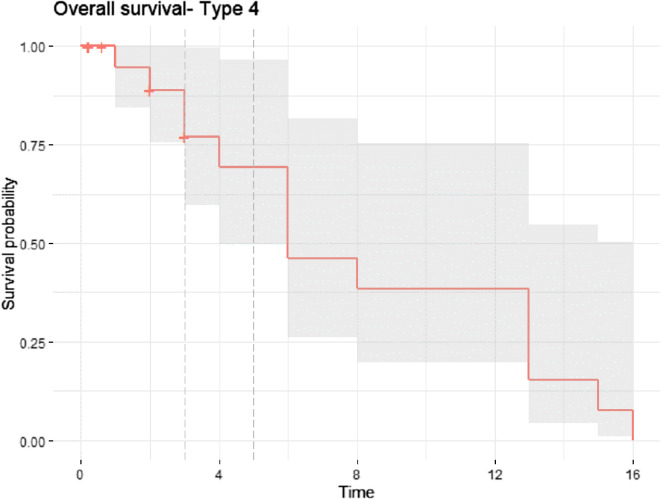
Kaplan–Meier curve for patient survival in individuals with Type 4 failure (infection). Five-year survival was reduced in this group: 77.0% at 3 years (95% CI: 59.6%–99.6%) and 69.3% at 5 years (95% CI: 49.9%–96.4%).

Patients without complications had a 5-year survival of 79.6%. In contrast, patients with complications showed lower survival, particularly those with Type 1 and Type 4 failures. For Type 4 complications, survival was 77.0% at 3 years (95% CI: 59.6–99.6%) and 69.3% at 5 years (95% CI: 49.9–96.4%) (Figure 4).

### Risk factors analysis

**Figure 5:**
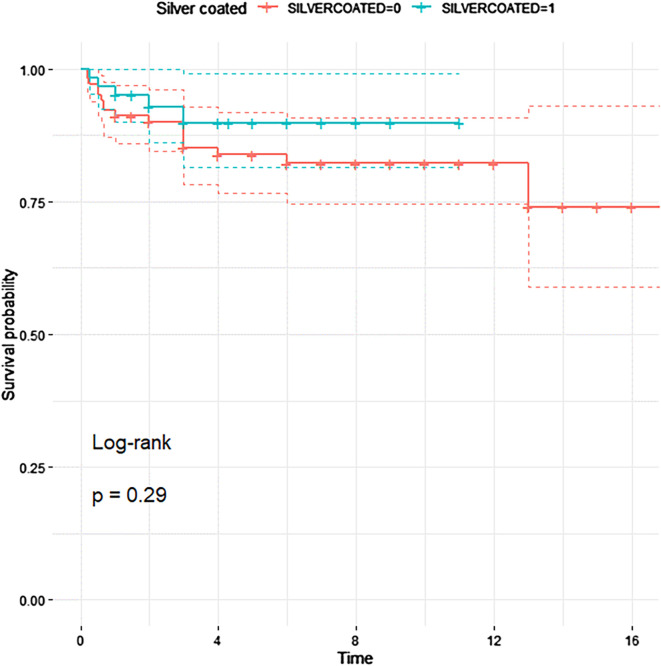
Kaplan–Meier curve comparing infection-free survival between silver-coated (cyan) and non-coated (red) implants. Although silver-coated implants showed a trend toward improved survival, the difference was not statistically significant (log-rank *p* = 0.29).

Risk factors for complications were analyzed, focusing on Types 1 and 4 due to adequate event frequency. Use of METS implants (Stanmore Implants Worldwide, United Kingdom) was independently associated with a significantly reduced risk of Type 1 failure (OR: 0.09, 95% CI: 0.00–0.45, *p* = 0.020), with an absolute soft-tissue failure rate of 3.5% compared to 15.7% for MUTARS implants (Implantcast GmbH, Germany). For Type 4 (infection), independent risk factors included prolonged hospitalization (OR: 1.09, 95% CI: 1.04–1.14, *p* < 0.001), higher ASA score (OR: 2.39, 95% CI: 1.32–4.32, *p* = 0.003), diabetes mellitus (OR: 6.32, 95% CI: 2.29–17.45, *p* < 0.001), increased body mass index (BMI) (OR: 1.25, 95% CI: 1.11–1.41, *p* < 0.001), prolonged operative time (OR: 1.02, 95% CI: 1.01–1.03, *p* < 0.001), and transfusion requirement (OR: 2.26, 95% CI: 1.32–3.89, *p* < 0.001). Although silver-coated implants showed a trend toward reduced infection rates (OR: 0.18, 95% CI: 0.03–1.15), the association did not reach statistical significance (*p* = 0.29) (Table 3, Figure 5).

**Table 3:** Multivariate analysis of risk factors for implant failure.

Failure type	Variable	Odds ratio (OR)	95% Confidence interval	*p*-value
Type 1	METS implant (vs. MUTARS)	0.09	0.00–0.45	0.020
Type 4	Prolonged hospitalization	1.09	1.04–1.14	<0.001
Type 4	Operative time	1.02	1.01–1.03	<0.001
Type 4	ASA score	2.39	1.32–4.32	0.003
Type 4	Diabetes mellitus	6.32	2.29–17.45	<0.001
Type 4	Increased BMI	1.25	1.11–1.41	<0.001
Type 4	Transfusion requirement	2.26	1.32–3.89	<0.001
Type 4	Silver-coated implant	0.18	0.03–1.15	0.29

Significant predictors of infection included prolonged hospitalization, elevated ASA score, diabetes mellitus, increased BMI, longer operative time, and transfusion requirement. Use of METS implants was protective against soft-tissue failure. Silver-coated implants demonstrated a non-significant trend toward infection risk reduction.

## Discussion

**Figure 6:**
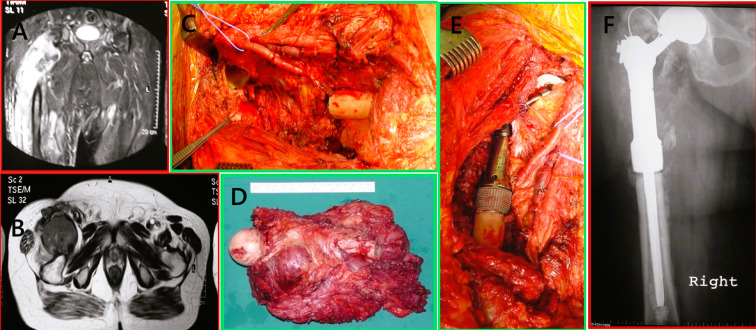
Preoperative imaging, intraoperative steps, and postoperative outcome of proximal femoral replacement in a 69-year-old male with recurrent high-grade leiomyosarcoma of the right thigh. (A) Coronal MRI demonstrating a recurrent, aggressive soft tissue mass infiltrating the proximal right femur with cortical destruction and intramedullary extension. (B) Axial MRI showing the extent of soft tissue involvement and medullary infiltration. (C) Intraoperative photograph following wide en bloc resection of the proximal femur. (D) Gross specimen showing the resected proximal femur with the attached high-grade leiomyosarcoma mass. (E) Intraoperative image during implantation of a modular Stanmore METS^®^ proximal femoral megaprosthesis. (F) Immediate postoperative anteroposterior radiograph of the right hip demonstrating final reconstruction with the Stanmore METS^®^ implant in situ.

**Figure 7:**
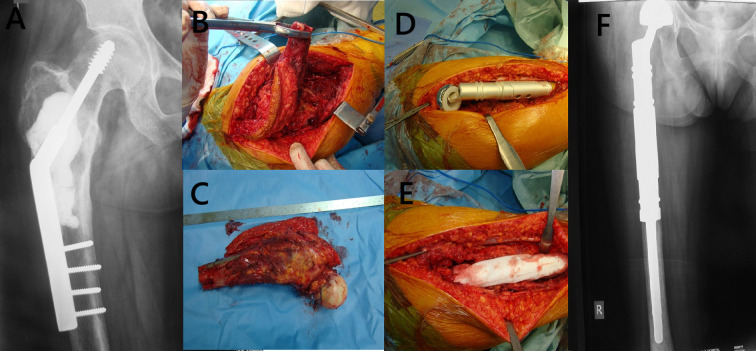
Preoperative imaging, intraoperative steps, and postoperative outcome of proximal femoral replacement using a MUTARS^®^ modular megaprosthesis (Implantcast GmbH) in a patient with recurrent Grade 2 chondrosarcoma of the right proximal femur. (A) Preoperative anteroposterior radiograph demonstrating a lytic lesion of the proximal right femur with pathological fracture and prior internal fixation using a dynamic hip screw. (B) Intraoperative image showing surgical exposure and mobilization of the tumor-bearing segment with removal of the previously implanted plate. (C) Gross specimen after wide en bloc resection of the proximal femur, including the tumor mass and attached hardware. (D) Intraoperative photograph showing implantation and alignment using the MUTARS^®^ modular megaprosthesis. (E) Reconstruction of periarticular soft tissues using a Trevira^®^ tube (Implantcast GmbH) for tendon reattachment and capsular stabilization. (F) Immediate postoperative anteroposterior radiograph showing the final position of the MUTARS^®^ proximal femoral replacement with appropriate alignment and fixation.

Megaprosthetic reconstruction is a well-established treatment for managing proximal femoral bone loss due to tumor resection or pathological fractures [7, 10, 11]. Its advantages include intraoperative modularity, immediate structural stability, and early postoperative function [12]. Since the 1970s, various implant designs have been developed, leading to a wide range of available systems today [2]. This study assessed the midterm outcomes of two widely used modular megaprosthesis systems – METS (Stanmore Implants Worldwide) and MUTARS^®^ (Implantcast GmbH, Germany) – in both oncologic and non-oncologic patients. Representative preoperative and postoperative radiographs of proximal femoral megaprosthetic reconstruction are presented in Figures 6 and 7, illustrating typical cases reconstructed with a METS implant (Figure 6) and a MUTARS^®^ implant (Figure 7). We focused on implant survival, failure modes, and risk factors for adverse outcomes.

Our study found 3-year and 5-year implant survival rates of 77.0% and 69.3%, respectively, consistent with previous reports [9, 10, 13–15]. These results are comparable to Bernthal et al. (56% at 20 years) but lower than short-term data from Nooh et al. and Gusho et al., who reported 92% survival at 5 years [8, 16, 17]. Discrepancies may be due to differences in patient populations, surgical indications, or follow-up duration. Nonetheless, both systems showed reliable midterm longevity.

Patient survival differed by indication, with those treated for metastatic lesions showing higher 5-year survival (75.3%) than those treated for primary bone tumors (64.2%). Interestingly, patients with metastatic disease demonstrated higher overall survival rates than those with primary bone tumors, which may appear counterintuitive. This observation likely reflects a selection bias, as patients with solitary or oligometastatic lesions – typically from indolent cancers such as renal or breast carcinoma, were selected for surgery based on favorable prognostic profiles. In contrast, patients with high-grade primary sarcomas often underwent wide resections in the context of more aggressive disease biology and poorer systemic outcomes. Additionally, adjuvant treatments for primary bone malignancies may have contributed to delayed complications or mortality unrelated to local reconstruction. These findings are consistent with studies showing better outcomes in oncologic cases compared to non-oncologic ones [18]. This highlights the need for individualized treatment strategies in limb salvage surgery.

The complication rate in our series was 30.9%, in line with previous studies [14, 15]. Soft tissue failures (Type 1) occurred in 11.5% of cases, similar to other studies [3, 9, 11]. Mechanical failure remains a significant concern, particularly due to loss of abductor function and large soft tissue resections [3, 18, 19]. The infection rate (13.3%) falls within the broad range reported in the literature (0–19.5%) and is linked to factors such as prolonged surgical time and blood transfusions [7, 13, 20]. These variables may reflect modifiable intraoperative and perioperative practices. Strategies such as reducing operative time through surgical planning and team coordination, enforcing strict sterile protocols, adopting restrictive transfusion thresholds, and preoperative patient optimization, particularly in cases involving diabetes or high BMI, may help mitigate this risk.

Our reoperation rate was 9.1%, consistent with prior reports [3, 15, 20], with infections, mechanical failure, and periprosthetic fractures being the most common causes. The relatively low revision and amputation rates (5.5%) suggest that careful planning and experienced surgical techniques can mitigate complications.

Multivariate analysis identified independent risk factors for infection, including diabetes, elevated BMI, higher ASA scores, longer surgical time, and blood transfusion [2]. This reinforces the importance of preoperative optimization to reduce infection risk. The use of silver-coated implants showed a non-significant trend toward reduced infection risk (OR = 0.18, *p* = 0.29), aligning with previous findings [21–23]. Further prospective studies are warranted.

Cemented fixation, used in 62.4% of cases, was protective against complications, particularly mechanical failure [24]. The decision to use cement was influenced by both patient age and indication. Cemented stems were more commonly used in elderly patients, those with poor bone quality, and in non-oncologic or palliative metastatic cases to allow for immediate stability and early mobilization. However, risks such as bone cement implantation syndrome should be considered, especially in frail patients [24]. Personalized fixation strategies remain crucial. A detailed comparison of implant survivorship, complication patterns based on the Henderson classification, and reoperation rates across key historical cohorts is presented in Table 4.

**Table 4:** Literature summary of proximal femoral replacement outcomes and complications (Henderson classification).

Reference	Patients	Follow-up (Years)	Cemented Femurs (%)	Implant Survival	Type 1 (%)	Type 2 (%)	Type 3 (%)	Type 4 (%)	Type 5 (%)	Reoperation Rate (%)
Morris et al., 1995 [25]	31	5.3	0%	100% (8 y)	3	0	0	3.2	6.5	9.7
Zehr et al., 1996 [26]	17	NR	100%	58% (10 y)	17	5.9	11.8	5.9	5.9	47
Unwin et al., 1996 [27]	263	3.8	100%	93.8% (10 y)	NR	3.4	1.9	2.6	4.6	6.1
Kabukcuoglu et al., 1999 [11]	54	9	100%	57% (20 y)	11.1	11.1	0	1.9	1.9	16.7
Donati et al., 2001 [21]	25	12.3	0%	NR	4	8	4	4.2	4	16
Ilyas et al., 2002 [28]	15	6.7	0%	NR	20	6.7	0	13.3	6.7	40
Ahlmann et al., 2006 [29]	96	4.9	100%	82% (5 y)	3.1	0	0	3.1	3.1	NR
Farid et al., 2006 [30]	52	10	100%	82% (10 y)	5.8	9.6	0	3.8	1.9	13.5
Menendez et al., 2006 [31]	96	1.5	100%	82% (10 y)	3.1	0	0	3.1	3.1	9.4
Gosheger et al., 2006 [14]	41	3.8	32%	79% (5 y)	7.3	NR	0	19.5	NR	NR
Orlic et al., 2006 [32]	44	9	0%	NR	9	0	NR	NR	NR	NR
Finstein et al., 2007 [33]	62	5	100%	62% (10 y)	4.8	11.3	4.8	4.8	7	19
Selek et al., 2008 [34]	45	NR	Mixed	NR	2.3	4.4	0	4.4	NR	6.7
Chandrasekar et al., 2009 [2]	100	1.3	100%	83% (5 y)	3	0	0	6	4	9
Potter et al., 2009 [35]	61	4.6	100%	93% (5 y)	6.6	3.3	0	4.9	0	9.8
Bernthal et al., 2010 [8]	86	5.4	100%	56% (20 y)	4.7	4.7	0	1.2	8.1	20.9
Henderson et al., 2011 [36]	403	NR	NR	NR	5.2	2.7	1	3	4	NR
Harvey et al., 2012 [13]	113	1.3	100%	100% (5 y)	9	0	0	9	NR	12.4
Pala et al., 2013 [37]	32	4.2	8.8%	58% (5 y)	9.4	0	0	9.4	9.4	NR
Calabró et al., 2016 [15]	109	2.5	50.5%	74% (9 y)	3.9	0	1	5.8	2.9	9.7
Houdek et al., 2016 [7]	204	7	100%	NR	7	3.9	NR	8	8	16
Johnson et al., 2019 [38]	26	6	100%	NR	7.7	0	0	15	3.8	31
Sørensen et al., 2019 [39]	26	NR	100%	NR	6	0	0	0	NR	15.4
Trovarelli et al., 2019 [9]	38	1.9	71%	82% (12 y)	10.5	2.6	0	2.6	2.6	18
Yilmaz et al., 2019 [40]	41	5	57%	NR	7.3	0	14.6	14.6	14.6	29
Bischel et al., 2020 [41]	45	1.4	NR	80% (6 y)	13.3	0	0	2.2	NR	11.6
Nooh et al., 2020 [17]	47	3.7	100%	92% (5 y)	8.5	4.3	0	4.2	10.6	NR
Gusho et al., 2021 [16]	19	0.7	NR	92% (5 y)	0	0	0	5	NR	5.3
Toepfer et al., 2021 [18]	58	4.4	Mixed	85% (10 y)	27.6	3.7	7.4	7.7	0	26
Sofulu et al., 2022 [42]	111	2	41.4%	97% (5 y)	1.75	1.8	0	2.6	2.7	6.1
Zavras et al., 2022 [3]	58	2.4	86%	88% (10 y)	12	0	9.8	1.7	NR	10.3
Zhang et al., 2022 [20]	16	3.9	0%	94% (7 y)	18.8	0	0	12.5	NR	12.5

This study has several limitations. The retrospective design introduces potential biases, and the relatively short follow-up for some patients limits our ability to assess long-term complications. Additionally, the heterogeneity of indications and surgical techniques may affect the generalizability of the results. Some associations – while clinically relevant – were accompanied by wide confidence intervals, suggesting potential underpowering due to limited subgroup sizes. Thus, caution is needed when interpreting long-term survival and failure rates. Future prospective and multicenter studies with standardized protocols are warranted to validate these findings, reduce institutional bias, and better characterize implant performance across different clinical contexts.

## Conclusion

This study confirms that proximal femoral megaprosthetic reconstruction remains a reliable option for limb salvage following tumor resection, offering acceptable midterm implant survival and complication profiles. Patient optimization, meticulous surgical technique, and vigilant postoperative management are essential to achieving long-term success.

## Data Availability

Data are available upon reasonable request, subject to privacy and ethical restrictions.
